# Functional Recovery and Safety of a Novel Hyaluronic Gel/Alginate Sheet for Complex Hand Injuries

**DOI:** 10.7759/cureus.96365

**Published:** 2025-11-08

**Authors:** Mitchell K Ng, Ira Lown

**Affiliations:** 1 Orthopedic Surgery, Rothman Orthopedic Institute, Philadelphia, USA; 2 Orthopedic Surgery, Austin Hand Group, West Lake Hills, USA

**Keywords:** flexor tendon repair, grip strength, hyaluronic acid/alginate hydrogel, patient-reported outcomes, postoperative adhesions, range of motion

## Abstract

Introduction: Adhesion formation following hand and microsurgical procedures is a relatively common but often substantial complication, potentially limiting mobility and impairing recovery. A bioresorbable hyaluronic acid (HA)/alginate hydrogel was developed to reduce this risk. This study aimed to 1) evaluate functional recovery after use of this novel hydrogel with grip strength and range of motion (ROM), 2) determine its effect on patient-reported outcomes and pain, and 3) assess its safety and complication profile.

Methods: A prospective, single-center cohort study was conducted in 17 patients (mean age: 43.5 years; range: 22-69) undergoing tendon repair, fracture fixation, or tenolysis. Intraoperatively, a novel HA/alginate hydrogel was applied circumferentially around the operative tendon. Patients were evaluated preoperatively and postoperatively (five days, 14 days, one month, three months, and six months). Primary outcomes included grip strength, ROM, Quick Disabilities of the Arm, Shoulder, and Hand (QuickDASH) scores, return-to-work status, and complications. Paired comparisons were performed between operative and contralateral extremities, with p < 0.05 considered significant.

Results: At three months, the mean grip strength was 55.5 ± 25.8 lbs in the operative hand compared with 68.9 ± 25.0 lbs in the contralateral hand. By six months, grip strength had improved to 64.0 ± 20.9 lbs, compared to 69.9 ± 20.6 lbs, with no significant difference. ROM deficits were most pronounced at the proximal interphalangeal joint early after surgery but improved steadily, with near-symmetric motion at six months. QuickDASH scores improved from 51.2 ± 24.3 at baseline to 16.1 ± 16.6 at six months (p < 0.01). One patient (5.8%) experienced early material extrusion that resolved without intervention. No infections or reoperations occurred.

Conclusion: Implementation of an HA/alginate sheet in complex hand surgery was safe and associated with favorable functional recovery and improved patient-reported outcomes. Larger randomized trials are warranted to confirm these preliminary findings.

## Introduction

Soft tissue adhesions following hand and microsurgical procedures remain a significant source of morbidity [1]. Dense scarring postoperatively can ultimately limit tendon excursion, tether nerves, and restrict joint motion, often leading to stiffness and potentially secondary surgery [2]. Reported rates of adhesions after flexor tendon repair approach 10%, and postoperative stiffness following plate fixation of phalangeal fractures has been noted in more than half of cases [3,4].

In response, there have been substantial efforts to reduce scarring, including management with pharmacologic agents, barrier membranes, and biologic grafts [5,6]. Human- and animal-derived products, such as amniotic membranes and collagen-based matrices, have demonstrated variable and inconsistent resorption, and in some cases, safety concerns secondary to adverse inflammatory responses or tendon rupture [5,7-9]. These shortcomings highlight the need for a biocompatible, resorbable material that can protect repaired structures during the critical early phase of healing without provoking foreign body reaction [10,11]. To this end, a fully resorbable hydrogel composed of hyaluronic acid (HA) and alginate was developed as a potential solution [11,12]. This novel material functions as a temporary gliding interface, reducing friction and permitting the diffusion of nutrients and growth factors [11,12]. It has been used in a wide range of soft tissue environments, such as tendons, ligaments, joints, peripheral nerves, and nearby vascular structures [13-16]. Notably, there have been promising results from early clinical use across several surgical fields, including, but not limited to, hand surgery, spine surgery, plastic surgery, foot and ankle surgery, and urological procedures [13-18]. However, to date, it has not been examined on a prospective, observational basis.

To this end, the purpose of this study was to assess outcomes in hand surgery, specifically 1) measuring functional recovery (via grip strength and range of motion (ROM)), 2) assessing associated patient-reported outcomes and pain, and 3) determining safety and any complications or adverse events. We hypothesized that intraoperative application of this novel HA/alginate hydrogel would be safe and that its bioresorbable composition would both support functional recovery and minimize adhesion-related complications.

## Materials and methods

Study design

We conducted a single-center observational cohort study to evaluate the safety and functional outcomes of a bioresorbable HA/alginate sheet (VersaWrap, Alafair Biosciences, Austin, Texas) used during hand and finger surgery. Institutional Review Board (IRB) approval for this study (WCG IRB Study No. 1336873; Protocol VW-001) was obtained on July 14, 2022, before patient enrollment. The study was conducted prospectively at a single site (Austin Hand Group, Austin, Texas) from August 2022 to August 2025, with IRB oversight formally closed on October 8, 2025.

All procedures were carried out by a fellowship-trained hand surgeon at a tertiary referral practice. IRB approval was obtained before patient enrollment, and written informed consent was secured from all participants. Patients between 18 and 70 years of age who required operative management of the hand or fingers (specifically tendon repair, fracture fixation, or tenolysis) were eligible. Participants were required to be capable of adhering to follow-up visits and study requirements. Patients were excluded if they were pregnant or breastfeeding, incarcerated, unable to communicate in English, or had known hypersensitivity to citrate, alginate, or hyaluronate. The operative surgeon also excluded patients with medical or psychosocial factors that might limit protocol compliance. Seventeen patients were enrolled (mean age: 43.5 years; range: 22-69 years; nine men; eight women). Fourteen of 17 (82.3%) patients worked steady jobs, with 10 of 17 (58.8%) working 40 hours per week (Table 1).

**Table 1:** Patient demographics F: female; M: male; MVA: motor vehicle accident; N/A: not applicable; ORIF: open reduction and internal fixation; L: left; R: right

Patient number	Injured finger	Dominant hand	Injury details	Age	Gender	Race	Ethnicity	Work baseline (Y/N)	Number of work hours (baseline)	Baseline work type
1	L index	L	Status post-ORIF with digital nerve neurolysis	58	M	Black	Non-Hispanic	Y	40	Moderate labor
2	R thumb	R	Fall while holding a metal pipe at work	35	M	White	Non-Hispanic	Y	40	Sedentary (desk job)
3	R index	R	Fall at home	66	F	Not reported	Not reported	N	N/A	N/A
4	R ring	R	MVA	26	M	Not reported	Not reported	Y	40	Moderate labor
5	R ring	R	MVA: hit by a car while riding a bike	56	F	Not reported	Not reported	Y	40	Moderate labor
6	L middle	R	Cutting meat with a knife	42	M	Black	Non-Hispanic	Y	40	Moderate labor
7	L little	R	Fall	55	F	White	Non-Hispanic	N	N/A	N/A
8	R little	R	Punching someone during an altercation	30	M	Not reported	Not reported	Y	40	Sedentary (desk job)
9	L little	L	Fall	33	F	White	Non-Hispanic	Y	40	Sedentary (desk job)
10	R thumb	R	Fall	46	F	White	Not reported	Y	25	Moderate labor
11	L thumb	R	Accident with a utility knife	69	M	White	Non-Hispanic	Y	40	Moderate labor
12	L index	R	Crushed by a metal sink cover	63	M	Not reported	Hispanic	Y	40	Heavy labor
13	R index	R	Cut with a knife	22	F	White	Hispanic	Y	12	Moderate labor
14	R thumb	R	Cut on glass	25	M	White	Hispanic	Y	30	Sedentary (desk job)
15	L little	R	MVA	30	F	White	Non-Hispanic	Y	30	Moderate labor
16	L middle	R	Caught in a dog leash	30	F	White	Not reported	Y	40	Sedentary (desk job)
17	R ring	R	Lifting a large appliance that slipped and sliced fingers	54	M	White	Non-Hispanic	N	N/A	N/A

Surgical technique and device application

At the end of the planned surgical procedure, VersaWrap was applied circumferentially around the repaired or mobilized tendon. The material was positioned directly over the tendon to act as a gliding interface with surrounding tissues. In accordance with its design, the sheet was applied without sutures or tissue adhesive. The specific surgical approach, fixation method, and tendon repair technique were left to the discretion of the treating surgeon, consistent with standard clinical practice.

Outcome measures

The primary outcome was postoperative functional recovery, measured by active and passive ROM of the affected digit(s). Secondary outcomes included grip strength, patient-reported outcomes using the Quick Disabilities of the Arm, Shoulder, and Hand (QuickDASH, QuickDASH© Institute for Work & Health, Toronto, ON, Canada, 2006. All rights reserved) questionnaire, and a visual analog scale for pain, wound healing, swelling, return-to-work status, and the incidence of adverse events or reoperations [19]. The QuickDASH was used under the free-use policy for noncommercial research, as outlined by the Institute for Work and Health. Patients were assessed before surgery, at the time of operation, and at five days, 14 days, one month, three months, and six months postoperatively. At each visit, wound status, ROM, pain, and QuickDASH scores were recorded. Grip strength testing was performed at the three- and six-month visits. Information regarding work status, ongoing therapy, and any additional procedures was also collected [19]. Adverse events were documented throughout follow-up, with severity and relationship to the device recorded by the investigator.

Statistical analyses

Continuous variables obtained for this study (e.g., grip strength, QuickDASH scores, and ROM) were characterized as means with standard deviations, whereas categorical variables (e.g., complications and return-to-work status) were reported as counts and percentages. Paired t-tests were conducted to compare the operative and contralateral hands, whereas the Wilcoxon signed-rank test was used for any nonparametric comparisons. Changes in functional scores and ROM across follow-up visits were evaluated utilizing analysis of variance. Statistical significance was defined as p < 0.05. As this study was structured as a pilot series, no a priori sample size calculation was performed. Additionally, enrollment reflected consecutive eligible cases treated during the study period. Tabulation and associated analyses were conducted using Microsoft Excel software (Microsoft, Redmond, Washington).

## Results

Functional recovery

Grip strength was tested at three and six months. At three months, mean grip strength measured 55.5 ± 25.8 lbs in the operative hand and 68.9 ± 25.0 lbs in the contralateral hand. By six months, strength improved to 64.0 ± 20.9 lbs in the operative extremity compared with 69.9 ± 20.6 lbs in the contralateral side. No statistically significant differences between the operative extremity and the contralateral side were identified at either three months (p = 0.88) or six months (p = 0.83) (Table 2).

**Table 2:** Postoperative grip strength at three and six months (in pounds) L: left; R: right; ND: no data

Patient number	Injured finger	Left-hand grip, 3 months	Right-hand grip, 3 months	Left-hand grip, 6 months	Right-hand grip, 6 months
1	L index	91	120	ND	ND
2	R thumb	84	79	84	79
3	R index	50	30	45	30
4	R ring	90	80	90	85
5	R ring	50	40	50	55
6	L middle	25	40	75	65
7	L little	40	65	50	60
8	R little	115	90	105	105
9	L little	40	45	45	45
10	R thumb	45	40	45	40
11	L thumb	65	65	80	85
12	L index	25	65	40	65
13	R index	50	30	55	50
14	R thumb	85	65	90	85
15	L little	ND	ND	65	60
16	L middle	85	65	60	70
17	R ring	ND	ND	105	80

Active ROM improved over time in the operative extremity. Deficits compared with the contralateral side were most apparent early but diminished with recovery (Table 3). At six months, most patients demonstrated near-symmetric motion between hands. Joint-level analysis showed that the proximal interphalangeal joint was the most limited in the early period, whereas metacarpophalangeal joint motion returned more quickly. When analyzed across all joints, although there was a trend toward decreased ROMs between operative and contralateral hands (p < 0.05), there were no statistically significant gains in the range of the operative hand at final follow-up (p < 0.05).

**Table 3:** Absolute difference in range of motion (in degrees) between operative and contralateral extremities DIP: distal interphalangeal joint; PIP: proximal interphalangeal joint; MCP: metacarpophalangeal joint; ND: no data; N/A: not applicable

Patient number	DIP	PIP	MCP
1	30-35	30-45	20-25
2	N/A	30-75	0-5
3	0	15	0-5
4	0-30	0-15	20-30
5	30-40	5-15	15-30
6	ND	5-20	20-35
7	ND	0-35	15-25
8	ND	0-15	0-30
9	ND	0-10	0-30
10	N/A	0-10	10-30
11	N/A	0-15	0-5
12	25-60	10-40	10-30
13	35-75	10-40	10-35
14	N/A	10-45	0-20
15	N/A	50-55	45-80
16	0	5-25	10-15
17	0-30	10-100	10-15

Patient-reported outcomes

**Figure 1:**
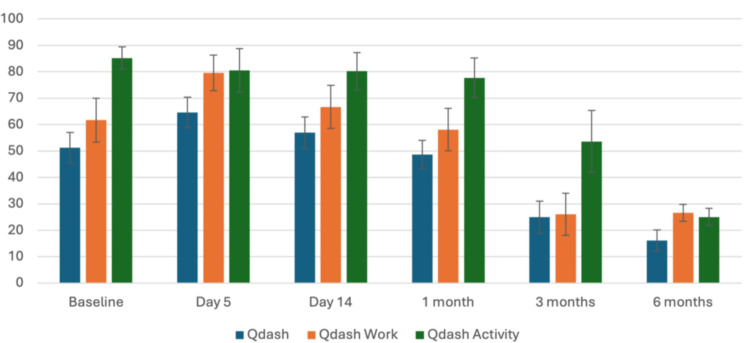
Postoperative QuickDASH scores QuickDASH: Quick Disabilities of the Arm, Shoulder, and Hand

QuickDASH scores demonstrated a consistent and statistically significant improvement over time from baseline to six months postoperatively (p < 0.01 for all). The mean baseline score was 51.2 ± 24.3, which increased at the five-day visit (64.6 ± 24.0), reflecting early postoperative disability. Scores then declined at each subsequent assessment: 56.9 ± 24.7 at 14 days, 48.6 ± 22.2 at one month, and 24.9 ± 25.0 at three months. By six months, the mean QuickDASH had fallen to 16.1 ± 16.6, indicating near-complete restoration of function for most patients. Work- and activity-related QuickDASH scores showed a parallel course, improving from baseline averages of 61.7 ± 34.1 and 85.2 ± 17.3 to 7.6 ± 13.0 and 12.5 ± 13.4, respectively, at six months. These changes are illustrated in Figure 1, using a bar chart highlighting the progressive decline in QuickDASH scores across all follow-up intervals.

Adverse events

VersaWrap was applied circumferentially around the repaired or mobilized tendon in all cases, without the use of sutures or adhesive. Wound healing was uncomplicated for nearly all participants. A single patient (5.8%) experienced extrusion of the material through the incision site during the early postoperative period. This resolved spontaneously at the first follow-up visit without further intervention. No infections, cases of erythema, or reoperations were observed.

## Discussion

This prospective cohort study examined the use of a bioresorbable HA/alginate hydrogel sheet in complex hand surgery. Overall, the material was safe and well tolerated, with no infections or reoperations observed and only a single episode of minor material extrusion that resolved promptly without treatment. Patients demonstrated steady functional recovery throughout. Grip strength approached that of the contralateral hand by six months, ROM deficits improved consistently throughout follow-up, and patient-reported QuickDASH scores declined significantly from preoperative levels. Taken together, our results suggest that this hydrogel is not only safe to apply but also provides a favorable environment for tendon healing and reduces the burden of adhesion-related stiffness.

Our first aim was to assess postoperative recovery of strength and motion. By the six-month visit, grip strength in the injured hand was nearly equal to that of the opposite side, and joint motion had largely normalized. These results compare well with prior reports showing persistent stiffness and incomplete recovery after tendon repair or phalangeal fracture fixation, conditions in which adhesions can be particularly problematic [4]. Published series note adhesion rates near 10% after flexor tendon repair and stiffness in more than half of patients after phalangeal fracture fixation [3,4,19]. Although our study did not include a control arm, the return to symmetric function in most patients supports the concept that this novel hydrogel serves as a temporary gliding surface, without impairing tissue healing.

Our second study objective was to evaluate postoperative patient-reported outcomes. QuickDASH scores improved in parallel with objective measures, improving from moderate disability preoperatively to near-normal function by six months. This trend reinforces the clinical relevance of the observed strength and motion gains, as patients themselves reported meaningful improvement in both daily activities and overall work capacity. A number of earlier studies have emphasized the negative impact of adhesions and stiffness on long-term function, making the consistent decline in disability scores in our cohort particularly notable [20-23]. A retrospective review of 126 patients undergoing flexor tendon repair found that, while most regained a fair amount of grip strength and improved QuickDASH scores (p < 0.05), 29% were still out of work six months postoperatively [24]. A randomized controlled trial of 45 patients undergoing flexor tendon repairs for zone 2 tendon injuries who received another hyaluronan gel found improved finger motion at all time intervals and higher return-to-work rates; however, it showed less relative improvement in QuickDASH scores compared with this study (20% relative to 68%, respectively) [25]. While we cannot separate the hydrogel’s specific contribution from the effects of surgery and therapy in this study, the favorable trajectory suggests that its use did not hinder, and may have supported, patient recovery.

The third aim of this study was to characterize the safety profile of this novel HA/alginate hydrogel prospectively. With no documented erythema, swelling, infections, wound complications, reoperations, and only one instance of early extrusion, which self-resolved, this novel HA/alginate hydrogel demonstrated a reassuring safety profile. This experience contrasts with prior reports of biologic barriers, such as amniotic membrane or collagen matrices, where variable resorption, inflammation, and even tendon rupture have been described [7,26].

There are several potential limitations to this study. First, our cohort included a relatively small number of patients from a single surgeon, limiting overall statistical power and external generalizability. Although this study was prospective, without a randomized comparison group, we are not able to establish superiority over conventional management without this hydrogel. Furthermore, follow-up was limited to six months and may not capture late complications or the durability of functional gains. Rehabilitation was standardized according to surgeon protocol but ultimately was not identical in intensity for all patients, introducing potential variability in outcomes. Larger, multicenter randomized studies with longer follow-up will be necessary to confirm these early observations. Nevertheless, this study is the first prospective observational study that evaluates and helps quantify the functional recovery seen in the use of a novel HA/alginate hydrogel during hand surgery.

## Conclusions

This prospective cohort study demonstrates that use of an HA/alginate hydrogel sheet in complex hand surgery cases is safe, quantifying functional recovery throughout and up to six-month follow-up. Improvements in grip strength, ROM, and patient-reported disability scores were consistent, and complications were rare, limited to a single case of early extrusion that resolved spontaneously. Although the absence of a control group and the small sample size limit definitive comparisons, our findings provide additional evidence supporting the use of this novel hydrogel as a potential adjunct to reduce adhesion-related morbidity. Further investigation through larger randomized trials with extended follow-up will be necessary to confirm the durability of these results and to more thoroughly characterize its surgical indications to more fully establish its role in routine practice.
